# Characterizing the Role of Glycogen Synthase Kinase-3α/β in Macrophage Polarization and the Regulation of Pro-Atherogenic Pathways in Cultured Ldlr^-/-^ Macrophages

**DOI:** 10.3389/fimmu.2021.676752

**Published:** 2021-07-30

**Authors:** Sarvatit Patel, Geoff Werstuck

**Affiliations:** ^1^Thrombosis and Atherosclerosis Research Institute, Hamilton, ON, Canada; ^2^Department of Chemistry and Chemical Biology, McMaster University, Hamilton, ON, Canada; ^3^Department of Medicine, McMaster University, Hamilton, ON, Canada

**Keywords:** atherosclerosis, glycogen synthase kinase-3α/β, macrophage polarization, macrophage function, inflammatory response, bone marrow derived macrophages, Classically activated macrophage, M1 and M2 macrophages

## Abstract

The molecular and cellular mechanisms that link cardiovascular risk factors to the initiation and progression of atherosclerosis are not understood. Recent findings from our laboratory indicate that endoplasmic reticulum (ER) stress signaling through glycogen synthase kinase (GSK)-3α/β induces pro-atherosclerotic pathways. The objective of this study was to define the specific roles of GSK3α and GSK3β in the activation of pro-atherogenic processes in macrophages. Bone marrow derived macrophages (BMDM) were isolated from low-density lipoprotein receptor knockout (Ldlr^-/-^) mice and Ldlr^-/-^ mice with myeloid deficiency of GSK3α and/or GSK3β. M1 and M2 macrophages were used to examine functions relevant to the development of atherosclerosis, including polarization, inflammatory response, cell viability, lipid accumulation, migration, and metabolism. GSK3α deficiency impairs M1 macrophage polarization, and reduces the inflammatory response and lipid accumulation, but increases macrophage mobility/migration. GSK3β deficiency promotes M1 macrophage polarization, which further increases the inflammatory response and lipid accumulation, but decreases macrophage migration. Macrophages deficient in both GSK3α and GSK3β exhibit increased cell viability, proliferation, and metabolism. These studies begin to delineate the specific roles of GSK3α and GSK3β in macrophage polarization and function. These data suggest that myeloid cell GSK3α signaling regulates M1 macrophage polarization and pro-atherogenic functions to promote atherosclerosis development.

## Introduction

Cardiovascular disease (CVD) is the leading cause of death in the world today (1) and atherosclerosis is a major underlying cause of CVD. Macrophages are centrally involved in every stage of the development of atherosclerosis, and they are the main cellular component within the atherosclerotic lesion (2, 3). Atherosclerosis initiates when endothelial cells (EC) respond to injury, which mediates the attachment and infiltration of monocytes. Monocytes invade the subintima and differentiate into macrophages. These macrophages take up modified-LDL particles and become foam cells, which form fatty streaks in the artery wall. Macrophage/foam cell apoptosis leads to the establishment of a necrotic core, which is a key feature of unstable plaques that are prone to rupture. Lesion rupture triggers atherothrombosis and can occlude the artery. This can lead to acute cardiovascular complications (myocardial infarction or stroke) and potentially death. The underlying molecular mechanisms that regulate macrophage function during the development of atherosclerosis are not completely understood.

Macrophages can be polarized into many different subtypes that have distinct characteristics and functions. The extreme phenotypes are pro-inflammatory (M1) macrophages and anti-inflammatory (M2) macrophages. M1 macrophages can be induced by exposure to T helper type 1 (Th1) cell products, such as interferon (IFN)-γ, or microbial products, such as lipopolysaccharide (LPS) (4). In contrast, M2 anti-inflammatory macrophages can be induced by exposure to T helper type 2 (Th2) cell products, including interleukin (IL)-4 or tumor growth factor (TGF)-β. M1 macrophages produce pro-inflammatory cytokines (TNFα, IL-1β) and are believed to promote atherosclerotic lesion development and complexity (5, 6), whereas M2 macrophages produce anti-inflammatory cytokines (IL-10) and have tissue remodeling properties (7, 8). Other macrophage subtypes have been identified, including M_ox_, M_hem_, and M4 (9). The roles and functions of these macrophages are less well understood. Macrophages are directly involved in a variety of processes during atherosclerosis including polarization, foam cell formation, apoptosis, cell viability/proliferation, and migration. The mechanism(s) and cellular signals that regulate macrophage polarization and other functions that contribute to the development of atherosclerosis are still unclear.

Glycogen synthase kinase 3 (GSK3) is a serine/threonine kinase that plays an important role in many cellular pathways that regulate metabolism and viability. GSK3 has been linked to several disorders and diseases, including cancer (10), bipolar mood disorder (11), diabetes (12), and Alzheimer’s disease (13). There are two main forms of GSK3 in mammals: GSK3α (51 kDa) and GSK3β (47 kDa), as well as the splice variant of GSK3β, GSK3β2 (14). Isoforms GSK3α and GSK3β are 98% homologous in the kinase domain and are expressed ubiquitously (15). GSK3α/β is predominantly located in the cytoplasm, endoplasmic reticulum (ER), and nucleus (16). GSK3α/β is a constitutively active kinase and its activity is directly regulated (inhibited) by the insulin and Wnt signaling pathways (17, 18). A study from our lab has shown that the presence of ER stress in Thp-1 derived macrophages activates the protein kinase R-like ER kinase (PERK) signaling branch of the unfolded protein response (UPR) to promote GSK3α/β activity (19). Recent evidence suggests that GSK3α and GSK3β have distinct functions (20–22). Whole-body GSK3α-deficient mice are viable and develop normally, while GSK3β deletion is embryonically lethal (23, 24). GSK3α and β play unique and independent roles in skeletal muscle cell insulin signaling (25–27), cardiomyocyte development and proliferation (23), and Th cell polarization (28). Recent studies support a role for GSK3α/β in atherosclerosis. Results from our lab suggest that myeloid deletion of GSK3α, pharmacological mitigation of ER stress (by 4 phenylbutyrate), or inhibition of GSK3α/β (by valproate) attenuates the progression of atherosclerosis (29–32). Together, these results suggest a role for myeloid-specific GSK3α in the development of atherosclerosis. However, the specific roles of myeloid GSK3α and GSK3β in macrophage polarization and other pro-atherogenic functions are not known.

In this study we have isolated bone marrow derived macrophages from myeloid cell-specific GSK3α and/or GSK3β-deficient Ldlr^-/-^ mice to characterize the roles of GSK3α and GSK3β in specific cellular functions. Our results demonstrate that GSK3α and GSK3β play distinctive roles in defining macrophage phenotype and regulating atherogenic responses.

## Materials and Methods

### Mouse Models

Myeloid-specific GSK3α- and/or GSK3β-deficient mice were created in an Ldlr^-/-^ genetic background, as previously described (30). Myeloid specific GSK3α knockout mice (*Ldlr^−/−^LyzMCre^+/-^GSK3α^fl/fl^ or* LMαKO), myeloid-specific GSK3β knockout mice (*Ldlr^−/−^LyzMCre^+/-^GSK3*β*^fl/fl^ or* LMβKO), and myeloid-specific GSK3α and β knockout mice (*Ldlr^−/−^LyzMCre^+/-^GSK3α^fl/fl^GSK3β^fl/fl^ or* LMαβKO) were utilized in this study. *Ldlr^−/-^GSK3α^fl/fl^GSK3β^fl/fl^* (Lαβfl/fl) mice were used as controls. All experimental mice had unlimited access to food and water and were maintained on a 12-hour light/dark cycle. All animal experiments were conducted with pre-approval of the McMaster University Animal Research Ethics Board. All experiments conform with the guidelines and regulation of the Canadian Council on Animal Care.

### Bone Marrow-Derived Macrophage Isolation and Polarization

At the age of 8-10 weeks, tibias and femurs were harvested and bone marrow was collected from Lαβfl/fl (control), LMαKO, LMβKO, and LMαβKO mice using a 70 μm nylon mesh passing through the medullary cavity. Cells were resuspended in Dulbecco’s Modified Eagle Medium (DMEM) containing 15% (v/v) fetal bovine serum, 100 IU/ml penicillin and 100 μg/ml streptomycin, 1X MEM non-essential amino acids, and 20 ng/ml macrophage colony stimulating factor (MCSF, Cell Signaling). Cells were counted using a hemocytometer and 5 x 10^6^ cells were seeded onto a 10 cm plate containing 10 ml of medium. After six days in a humidified 37°C incubator (5% CO2), cells were washed twice with warm, sterile 1X Dulbecco’s phosphate-buffered saline (DPBS) without calcium or magnesium. Macrophages were detached using accutase (Cedarlane), and cells were counted with a hemocytometer and replated for subsequent experiments. Subsets of cells were polarized to M1 macrophages by exposure to 10 ng/ml lipopolysaccharide (LPS), or M2 macrophages by exposure to 10 ng/ml IL-4 for 24 hours or left unstimulated as M0 macrophages (30, 33). Culture media was collected after the 24 hours of treatment (UT, LPS or IL-4) and cytokine and chemokine levels were quantified using the pro-inflammatory focused multiplexing LASER Bead Assay ([Mouse Cytokine Array/Chemokine Array 31-Plex (MD31), Eve Technologies, Calgary, AB].

### Flow Cytometry

Plated cells were detached with accutase and incubated with an Fc-receptor blocking antibody (anti-CD16/32 (1:100), eBioscience) for 30 min. Macrophage-specific surface markers were identified by incubating with fluorescently labeled antibodies against CD11b (1:50, Life Technologies) and F4/80 (1:50, BD Pharmagen) for 1 hour. Unbound antibodies were washed off in FACS buffer (PBA, 0.1% BSA, 0.1% sodium azide). Flow cytometry was performed using a BD LSR II flow cytometer (BD Biosciences).

### Gene Expression

BMDM were seeded onto 12-well tissue culture plates at a density of 4 x 10^5^ cells/well in 1 ml medium and polarized as described above. Total RNA was isolated using TRIzol^®^ Reagent (Invitrogen), as previously dscribed (19, 30). Purified total RNA was resuspended in DNase/RNase-free water and RNA concentration and purity were determined using a Nanodrop spectrophotometer (Thermo Fisher Scientific). DNA was prepared from 1 μg of total RNA using the High-Capacity cDNA Reverse Transcription Kit (Applied Biosystems). Quantitative real-time reverse transcription-polymerase chain reaction (RT-PCR) was performed using 1 μl of resulting cDNA, 12.5 μl SensiFAST SYBR-Rox (Thermo Fisher Scientific), 1.25 μl of forward and reverse primers (500 nM, IDT) (**Supplementary Table I**), and 8 μl of RNase-water in a total volume of 24 μl/well. The following conditions were used to amplify cDNA: 10-minute hold at 95°C, followed by 40 cycles consisting of a 15-second melt at 95°C, followed by 1-minute annealing at 60°C. Relative quantitative analysis (2-ddCt) was performed by normalizing data to the β-actin reference gene.

### Characterization of Macrophages

BMDM were cultured and resuspended in lysis buffer (4x SDS PAGE sample buffer). Cell extracts were fractionated by SDS-PAGE and transferred to a polyvinylidene difluoride (PVDF) membrane (Bio-Rad). The membranes were blocked with 5% non-fat milk in TBST (10 mM Tris, pH 8.0, 150 mM NaCl, 0.5% Tween 20) for 45min and then incubated overnight with primary antibody against GSK3α/β (1:1000, Cell signaling) or β-Actin (1:3000, Sigma) at 4°C. Membranes were washed and incubated with a horseradish peroxidase-conjugated anti-rabbit antibody (1:200, Dako) or anti-mouse antibody (1:200, Dako) for 1 h. Blots were washed with TBST three times for 5 min each and developed with the ECL system (Millipore). Images were captured using a Molecular Imager ChemiDoc XRS+ (Bio-Rad).

### AlamarBlue Cell Viability Assay

BMDM were cultured in 96 well tissue culture plates at a density of 1 x 10^5^ cells/well/100 μl medium. Cells were polarized as described above and then treated with 10 μM thapsigargin (Tg), or 10 μg/ml tunicamycin (Tm), or left untreated for 24 hours. Cell viability was determined using the alamarBlue™ assay (Bio-rad). Cells were washed and alamarBlue™ reagent (Bio-Rad) was added. Absorbance was determined at 570 nm (reduction) and 600 nm (oxidation) to calculate cell viability.

### Immunofluorescent Staining

BMDM were seeded onto 8 chamber slides (Thermo Fisher Scientific) at a density of 1 X 10^5^ cells/200ul/chamber and incubated at 37°C. Immunostaining was performed as previously described (19, 30). Cells were washed with 1x PBS, fixed with 4% paraformaldehyde (PFA) for 15 min. Cells were permeabilized with 0.5% Triton X-100 for 5 min and then incubated in blocking solution (3% goat serum, 0.5% BSA, 1X PBS) for 1hr. Primary antibodies against the proliferation marker Ki67 (1:200, Abcam), NF-κB p65 (1:50, Santa Cruz), NLRP3 (1:50, Abcam), or CCR7 (1:100, Abcam) were added. After 24 hours, cells were washed and then incubated with secondary antibodies, Alexa Fluor 488 goat anti-mouse IgG (1:250, Thermo Fisher Scientific) or Alexa Fluor 488 goat anti-rabbit IgG (1:250, Thermo Fisher Scientific), for 2 hrs. Separate slides of cells were stained with pre-immune IgG instead of primary antibodies to control for non-specific staining (**Supplementary Figure I**). Cells were washed and stained with 4′,6-diamidino-2-phenylindole dihydrochloride (DAPI, 1:5000, Invitrogen). The slides were mounted using Fluoromount Aqueous Mounting Medium (Sigma) and stored at 4°C in the dark. Images of the stained sections were collected using a Leitz LABORLUX S microscope connected to a DP71 Olympus camera. ImageJ 1.52q software was used to quantify immunofluorescent staining. For each experimental group of cells four biological replicates were analyzed. For each replicate, a minimum of four images were captured, each containing approximately 200 cells. The stained area over background as well as cell number were quantified using ImageJ 1.52q software. Data from each image of a biological replicate (four images) were combined providing a stained area per cell with a minimum of 800 cells. Data are presented as average stained area per cell from four biological replicates.

### Oil Red O Staining

Chamber slides with 8 chambers (wells) were used for the lipid accumulation assay. Cells were plated in 8 chamber slides (Thermo Fisher Scientific) at a density of 1.5 X 10^5^ cells/200ul/chamber and incubated at 37°C for 24 hrs in DMEM. Oil Red O stock solution was prepared by dissolving 2.5 g Oil Red O powder (Sigma) in 500 ml isopropanol (100%) in a water bath at 70°C for 10 min. The stock solution was filtered while it was warm. Before staining, the working solution was prepared by diluting stock solution 3:2 within ddH_2_O. Cells were fixed with 4% PFA for 15 min. and permeabilized with 0.5% Triton X-100 for 5 min. Cells were stained with filtered oil red O working solution at 37°C for 15-20 min. Cells were washed in 60% isopropanol for 15-30 sec. and PBS 2 times, then stained with DAPI (1:5000, Invitrogen) for 2-5 min. The cells were mounted by using Fluoromount Aqueous Mounting Medium (Sigma) and stored at 4°C in the dark. Lipid content was visualized by a bright-field or fluorescent microscope (Olympus BX41 microscope connected to a DP71 Olympus camera) and quantified. For each experimental group of cells, four biological replicates were analyzed. For each replicate, four images were captured, each containing x approximately 200 cells. The stained area over background as well as cell number were quantified using ImageJ 1.52q software. Data from each image of a biological replicate (four images) were combined providing a stained area per cell with a minimum of 800 cells. Data shown are average stained areas per cell from four biological replicates.

### Migration Assay

Cells were seeded at a density of 0.8 X 10^5^ cells/200ul/insert onto Transwell inserts (pore size of 3 µm, Corning Costar) that were pre-coated with rat tail collagen I (4mg/ml, Millipore). Cells were added in the upper chamber and incubated at 37°C for 1 hr in serum-free media. These filter inserts were placed in wells containing the serum-free media with 0.5ug/ml chemokine ligand 19 (CCL19, R&D Systems) and incubated at 37°C. After 4 hrs, inserts with the cells were removed and washed with 1x PBS. Cells were fixed with 4% PFA for 15 min. After washing with 1x PBS, cells were stained with DAPI (1:5000, Invitrogen) for 2-5 min. Filters were then rinsed twice with 1x PBS. The cells on the upper surface, that had not migrated, were removed by carefully scraping with a cotton swab. Migrated cells, on the lower surface, were visualized and quantified using a fluorescent microscope (Olympus BX41 microscope connected to a DP71 Olympus camera, with 4x objective). For each experimental group of cells four biological replicates were analyzed. For each replicate, a minimum of five images were captured. The number of cells number was quantified using ImageJ 1.52q software. Data from each image of a biological replicate (five images) were combined providing a total number of cells migrated. Data shown are average percentage migrated cells from four biological replicates.

### Extracellular Flux Analysis

BMDM were plated in Seahorse XF24 plate (Agilent) at a density of 40,000/well in DMEM and cultured for 24 hours before the medium was replaced with a fresh DMEM. The assay was performed as described previously (34). One hour before the assay, media were exchanged for XF24 media. Mito Stress assay was performed by sequential addition of Oligomycin (inhibitor of ATP synthesis), carbonyl cyanide 4-(trifluoromethoxy) phenylhydrazone (FCCP, uncoupling agent), and rotenone/antimycin A (inhibitors of complex I and complex III of the respiratory chain, respectively) were diluted into XF24 media and loaded into the accompanying cartridge to achieve final concentrations of 1 μM, 2 μM, and 0.5 μM, respectively. Injections of the drugs into the medium occurred at the time points specified. Oxygen consumption rate (OCR) and extracellular acidification rate (ECAR) were monitored using a Seahorse Bioscience XF24 Extracellular Flux Analyzer (Agilent). Data are analyzed using Wave Desktop Software.

### Statistical Analysis

All statistical analyses were performed using GraphPad Prism 8 software. Data were analyzed by one- or two-way ANOVA, followed by the Bonferroni multiple comparison test between all groups. Error bars represent the standard error of the mean (SEM). For all experiments, a *p*-value lower than 0.05 was considered statistically significant. *p<0.05, **p<0.01, ***p<0.001, ****p<0.0001.

## Results

### Characterization of Myeloid Cell-Specific GSK3α and/or GSK3β Deficiency in Ldlr^−/−^ Mice

**Figure 1:**
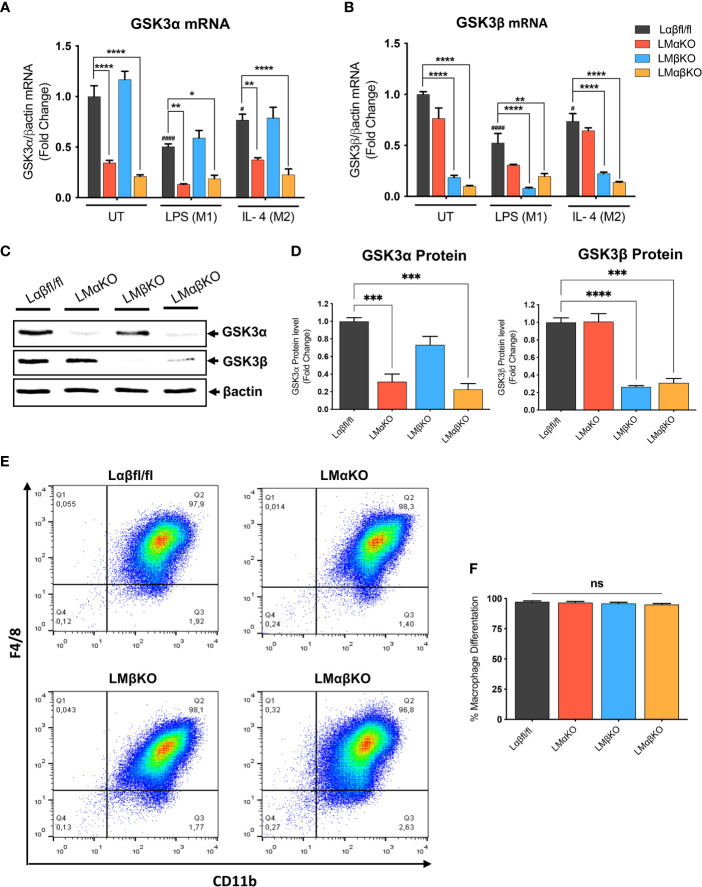
Effect of GSK3α and/or GSK3β deficiency on macrophage differentiation and characterization of myeloid cell–specific GSK3α and/or GSK3β knockout in BMDM. BMDM isolated from Lαβfl/fl(control), LMαKO, LMβKO and LMαβKO mice. BMDMs were exposed to 10 ng/mL LPS or 10 ng/mL IL-4 for 24 hours to induce M1 or M2 macrophage polarization respectively. Quantification of **(A)** GSK3α and **(B)** GSK3β gene expression in BMDM by RT-PCR. Data are normalized to the βactin reference gene. **(C)** Whole tissue lysates from control and macrophage specific GSK3α and/or GSK3β knockout mice were resolved by SDS-PAGE and probed with antibodies against GSK3α, GSK3β and βactin. Representative images are shown (full-length blots are presented in **Supplementary Figure II**) **(D)** Quantified GSK3α and GSK3β protein levels in BMDM determined by densitometry analysis. To determine the bone marrow progenitor cells differentiation into macrophages, cells were labelled with antibodies against the macrophage-specific surface markers, CD11b and F4/80. Cells from each experimental group were examined on a BD FACS calibur flow cytometer. **(E)** Contour diagram of CD11b/F4/8 of BMDM. **(F)** Comparison of number of macrophages differentiated in various groups. Results are reported as the fold change relative to control UT. n=3-4; mean ± SEM; # is the comparison to Control UT; * is the comparison between control and KOs (within same treatment); ^#^p < 0.05, ^####^p < 0.0001, *p < 0.05, **p < 0.01, ***p < 0.001, ****p < 0.0001. GSK3 indicates glycogen synthase kinase 3; BMDM, bone marrow derived macrophages; UT, untreated; LPS, lipopolysaccharide; IL-4, interleukin-4; ns, no-significant difference.

Myeloid GSK3α and/or GSK3β deletion did not significantly alter the number of monocytes or other cell types in whole blood (**Supplementary Table II**). To determine the effect of GSK3α and/or GSK3β deficiency on macrophage function, bone marrow was harvested from 8-10-week-old Ldlr^-/-^ mice with myeloid-specific GSK3α and/or GSK3β deficiency. Bone marrow was cultured in the presence of MCSF to induce macrophage differentiation. Subsets of BMDM were exposed to 10 ng/ml LPS or 10 ng/ml IL-4 for 24 hrs to polarized them to M1 and M2, respectively. Myeloid cell-specific deficiency of GSK3α and/or GSK3β was confirmed by RT-PCR and immunoblot. Polarization of BMDM to either M1 (LPS) or M2 (IL4) resulted in a significant decrease in gene expression of GSK3α and GSK3β in both control and specific knockout macrophages (**Figures 1A, B**). There was no detectable compensation in the gene expression (**Figures 1A, B**), or protein expression (**Figures 1C, D** and **Supplementary Figure II**), of the retained homolog when GSK3α or GSK3β was deleted.

To determine the efficiency of MCSF-induced differentiation, the macrophage (CD11b+ F4/80+ cells) number was assessed by flow cytometry. Results show that over 95% of cells were F4/80+ and CD11b+ (**Figure 1E** and **Supplementary Figure III**). Our gating method was able to capture all of CD11b+ and F4/80+ cells in one box. The results show that bone marrow from LMαKO, LMβKO, and LMαβKO mice is not significantly different than Lαβfl/fl (control) bone marrow in terms of its ability to differentiate into macrophages (**Figure 1F**). This suggests that GSK3α and/or β deficiency does not affect bone marrow cell number or the efficiency of bone marrow differentiation into M0 macrophages.

### GSK3α Deficiency Impairs, and GSK3β Deficiency Enhances, Inflammatory Macrophage Polarization

**Figure 2:**
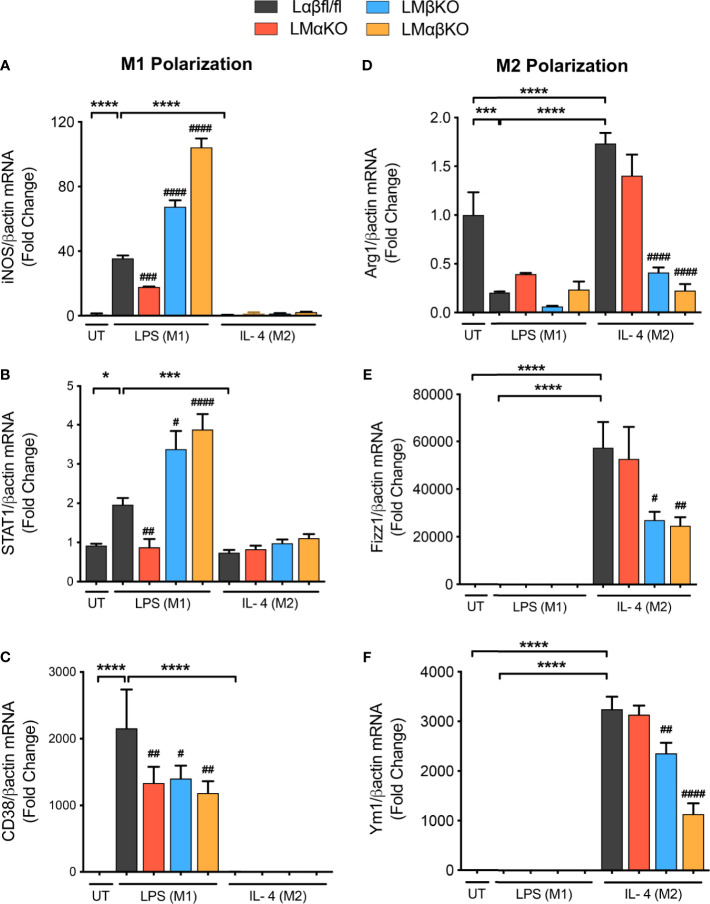
M1 macrophage polarization is impaired with a GSK3α deficiency and enhanced with a GSK3β deficiency. BMDMs were isolated from Lαβfl/fl(control), LMαKO, LMβKO and LMαβKO mice and exposed to 10 ng/mL LPS or 10 ng/mL IL-4 for 24 hours to induce M1 or M2 macrophage polarization respectively. Polarization efficiency were examined by quantify the transcription expression of the gene associated with M1 and M2 macrophage polarization by using RT-PCR. M1 Markers: **(A)** iNOS **(B)** STAT1 **(C)** CD38 and M2 Markers: **(D)** Arg1 **(E)** Fizz1 **(F)** Ym1. Data are normalized to the βactin reference gene. Results are reported as the fold change relative to control UT. n=3-4; mean ± SEM; * is the comparison between UT, LPS(M1), and IL-4(M2) treatments; # is the comparison between control and KOs (within same treatment); ^#^p < 0.05, ^##^p < 0.01, ^###^p < 0.001, ^####^p < 0.0001, *p < 0.05, ***p < 0.001, ****p < 0.0001. GSK3 indicates glycogen synthase kinase 3; BMDM, bone marrow derived macrophages; UT, untreated; LPS, lipopolysaccharide; IL-4, interleukin-4; iNOS, inducible nitric oxide synthases; STAT1, signal transducer and activator of transcription 1; CD38, cluster of differentiation 38; Arg1, arginase 1; Fizz1, found in inflammatory zone 1.

To examine the effect of GSK3α and/or GSK3β deletion on macrophage polarization, subsets of BMDM were exposed to 10 ng/ml LPS or 10 ng/ml IL-4 for 24 hrs. Polarization efficiency was determined by analyzing gene expression of inducible nitric oxide synthase (iNOS), signal transducer and activator of transcription 1 (STAT1) and cluster of differentiation 38 (CD38) as markers of inflammatory (M1) macrophages (**Figures 2A–C** and **Supplementary Figure IVA**); and arginase 1 (Arg1), found in inflammatory zone 1 (Fizz1) and Ym1 as markers of anti-inflammatory (M2) macrophages (**Figures 2D–F** and **Supplementary Figure IVB**). As expected, exposure of Lαβfl/fl (control) macrophages to LPS promotes iNOS, STAT1 and CD38 expression, and exposure to IL-4 promotes Arg1, Fizz1 and Ym1 expression. LMαKO macrophages exhibit impaired LPS-induced iNOS, STAT1 and CD38 expression. LMβKO and LMαβKO macrophages show significantly increased LPS-induced iNOS and STAT1 expression and decreased IL-4-induced Arg1, Fizz1 and Ym1 expression. LMβKO and LMαβKO macrophages show significantly decreased LPS-induced CD38 expression. LMαβKO macrophages treated with LPS increased genes expression of heme oxygenase (HO1) and Txnd1 (M_ox_ markers) compared to the Lαβfl/fl (control) (**Supplementary Figure IVC**). These results suggest that GSK3α and GSK3β play a central role in M1 and M2 polarization, respectively.

### Pro-Inflammatory Response Is Impaired in GSK3α-Deficient, and Enhanced in GSK3β-Deficient, Macrophages

**Figure 3:**
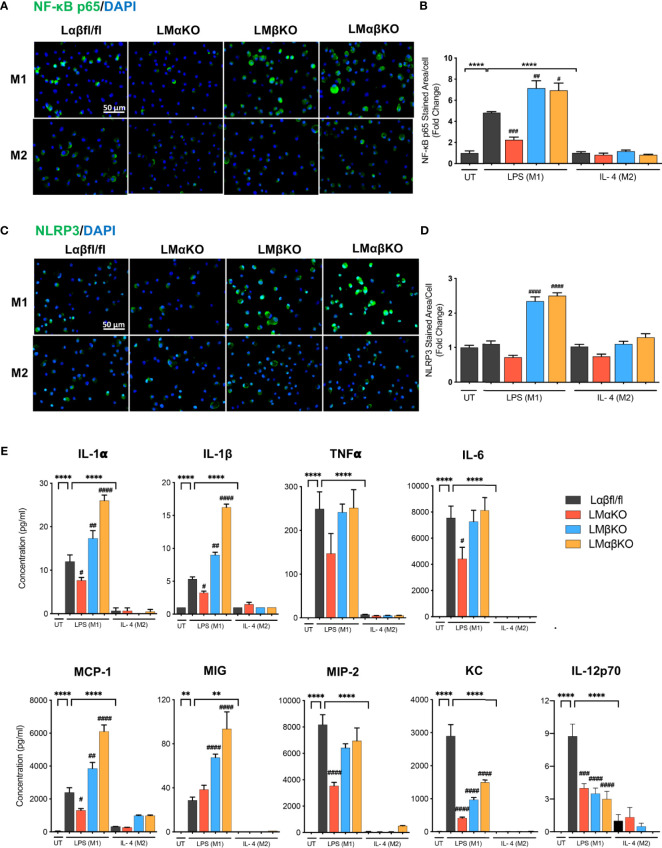
Pro-inflammatory response is impaired with GSK3α deficiency and enhanced with GSK3β deficiency. Pro-inflammatory response was examined by quantify the expression of the NF-κB and NLRP3. NF-κB and NLRP3 were immunostained using primary antibody, anti-NF-κB p65 or anti-NLRP3. Representative images of **(A)** NF-κB p65 and **(C)** NLRP3 staining of control, GSK3α and/or GSK3β-deficient macrophages. **(B)** NF-κB p65 and **(D)** NLRP3 stained area per cell was quantified. Results are reported as the fold change relative to control UT. Results are reported as the fold change relative to control UT. **(E)** Concentration of pro-inflammatory cytokines (IL-1α, IL-1β, TNFα, IL-6, IL-12p70) and chemokines (MCP-1, MIG, MIP-2, KC) in culture media were determined by mouse cytokine and chemokine array after 24 hours of UT, LPS(M1) or IL-4(M2) treatment. n=3-4; mean ± SEM; * is the comparison between UT, LPS(M1), and IL-4(M2) treatment; # is the comparison between control and KOs (within same treatment); ^#^p < 0.05, ^##^p < 0.01, ^###^p < 0.001, ^####^p < 0.0001, **p < 0.01, ****p < 0.0001. GSK3; glycogen synthase kinase 3; BMDM, bone marrow derived macrophages; UT, untreated; LPS, lipopolysaccharide; IL-4, interleukin-4; NF-κB, nuclear factor kappa-light-chain-enhancer of activated B cells; NLRP3, NLR family pyrin domain containing 3; TNFα, tumour necrosis factor; MCP-1, monocyte chemoattractant protein-1; MIG, monokine induced by gamma interferon; MIP-2, macrophage inflammatory protein-2.

The inflammatory response was determined by analyzing the expression of nuclear factor kappa-light-chain-enhancer of activated B cells (NF-κB) and NLR family pyrin domain containing 3 (NLRP3) in BMDM by immunofluorescence staining (**Figures 3A, C**). BMDM from Lαβfl/fl (control), LMαKO, LMβKO, and LMαβKO mice were cultured for 48 hrs. Macrophages were fixed and stained with antibodies against NF-κB p65 or NLRP3 and DAPI. Immunofluorescence was quantified (**Figures 3B, D**). As expected, M1 macrophages have enhanced NF-κB expression, relative to M2 macrophages. Results suggest that LMαKO M1 macrophages have decreased expression of NF-κB (**Figures 3A, B**). LMβKO and LMαβKO M1 macrophages showed an increase in the expression of NF-κB and NLRP3 (**Figures 3B, D**). Furthermore, LMαβKO macrophages showed significantly increased gene expression of CCAAT Enhancer Binding Protein Beta (C/EBPβ) in comparison to Lαβfl/fl (control) (**Supplementary Figure V**). Levels of the pro-inflammatory cytokines including IL-1α, IL-1β, TNFα, IL-6, IL-12p70 and chemokines including monocyte chemoattractant protein-1(MCP-1), monokine induced by gamma interferon (MIG), macrophage inflammatory protein-2 (MIP-2), KC were determined using Mouse Cytokine Array/Chemokine Array 31-Plex (MD31) (Eve Technologies; Canada). Levels of the anti-inflammatory cytokines including IL-10 and IL-4 were also determined using the same method (**Supplementary Figure VI**). LMαKO macrophages showed decreased expression of IL-1α, IL-1β, TNFα, IL-6, IL-12p70, MCP-1, MIP-2, and KC compared to Lαβfl/fl (control) (**Figure 3E**). LMβKO and LMαβKO macrophages showed increased expression of IL-1α, IL-1β, MCP-1 and MIG compared to Lαβfl/fl (control) (**Figure 3E**). LMβKO and LMαβKO macrophages showed decreased expression of IL-12p70 and KC compared to Lαβfl/fl (control) (**Figure 3E**). Together these results suggest that GSK3α deficiency impairs the pro-inflammatory response and GSK3β deficiency enhances the pro-inflammatory response of M1 macrophages.

### Lipid Accumulation Is Impaired in GSK3α-Deficient Macrophages and Increased in GSK3β-Deficient Macrophages

**Figure 4:**
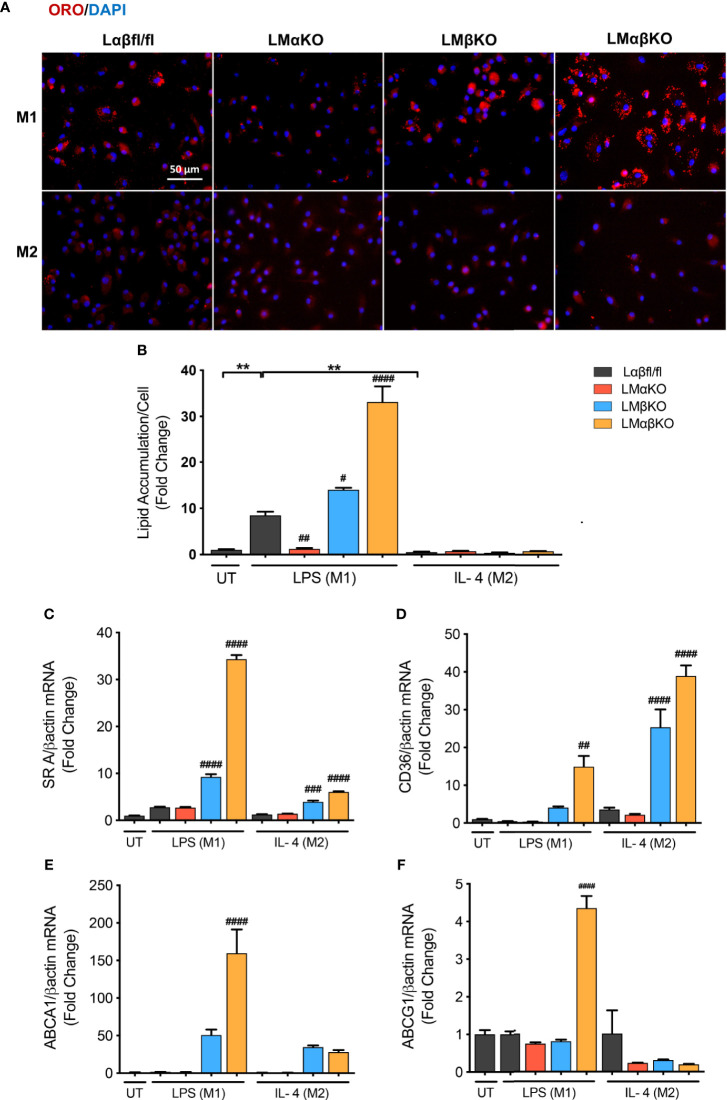
M1 Macrophage lipid accumulation is impaired by GSK3α deficiency and enhanced with GSK3β deficiency. Lipid accumulation of these macrophages were determined by Oil Red O staining. **(A)** Oil red O staining of GSK3α- and/or GSK3β-deficient macrophages and lipid accumulation was quantified by measuring **(B)** ORO-stained area/celI. Lipid accumulation were also measure by determining gene expression of lipid transport proteins such as **(C)** SR A **(D)** CD36 **(E)** ABCA1 **(F)** ABCG1. Results are reported as the fold change relative to control UT. n=3-4; mean ± SEM; * is the comparison between UT, LPS(M1), and IL-4(M2) treatments; # is the comparison between control and KOs (within same treatment); ^#^p < 0.05, ^##^p < 0.01, ^###^p < 0.001, ^####^p < 0.0001, **p < 0.01. GSK3 indicates glycogen synthase kinase 3; ORO, oil red o; UT, untreated; LPS, lipopolysaccharide; IL-4, interleukin-4; SR A, scavenger receptor A; ABCA1, ATP-binding cassette transporter; ABCG1, ATP Binding Cassette Subfamily G Member 1.

Macrophages are endocytotic cells that readily take up lipoprotein particles and cell debris. To determine the roles of GSK3α and GSK3β in lipid accumulation, BMDM were isolated from Lαβfl/fl (control), LMαKO, LMβKO, and LMαβKO mice and cultured for 24 hrs. Cells were stained with Oil Red O and analyzed for lipid accumulation by quantifying ORO-stained area. The results indicate that M1 macrophages accumulate significantly more lipid compared to M2 macrophages (**Figures 4A, B** and **Supplementary Figure VIIA**). In addition, LMαKO macrophages have a decreased tendency to accumulate lipids while LMβKO and LMαβKO macrophages have an increased ability to accumulate lipids (**Figure 4B**).

We next investigated the effect of GSK3α and/or GSK3β deletion on gene expression of scavenger receptor (SR)-A and CD36, two genes encoding proteins involved in lipid uptake (**Figures 4C, D**), and ATP-binding cassette transporter (ABCA1) and ATP binding cassette subfamily G member 1 (ABCG1), two genes encoding proteins involved in lipid efflux (**Figures 4E, F**). Results show that there is an increase in gene expression of (SR)-A, CD36, and ABCA1 in LMβKO and LMαβKO M1 macrophages. LMαβKO M1 macrophages displayed increased gene expression of ABCG1. LMβKO and LMαβKO M2 macrophages show a significant increase in (SR)-A and CD36 expression that does not affect actual lipid accumulation in these experiments. There is no difference in gene expression of these markers in LMαKO macrophages, relative to controls. Analysis of other genes involved in lipid accumulation, including SR-B1, liver X receptor alpha (LXRα), and lecithin–cholesterol acyltransferase (LCAT), revealed no significant differences in expression in LMαKO, LMβKO or LMαβKO macrophages compared to control (within the same treatment group) (**Supplementary Figure VIIB-VIID**). The effect of GSK3α and/or GSK3β deficiency on lipid biosynthesis was determined by analyzing the gene expression of 3-hydroxy 3-methylglutaryl-CoA (HMG-CoA) and fatty acid synthase (FAS). Results show that LMβKO and LMαβKO macrophages have significantly reduced gene expression of HMG-CoA and FAS (**Supplementary Figures VIIE, VIIF**). Together, these findings suggest that the presence of GSK3α promotes lipid uptake and accumulation and GSK3β impedes lipid accumulation in M1 macrophages.

### GSK3α and GSK3β Play Redundant Roles in Cell Viability and Proliferation

**Figure 5:**
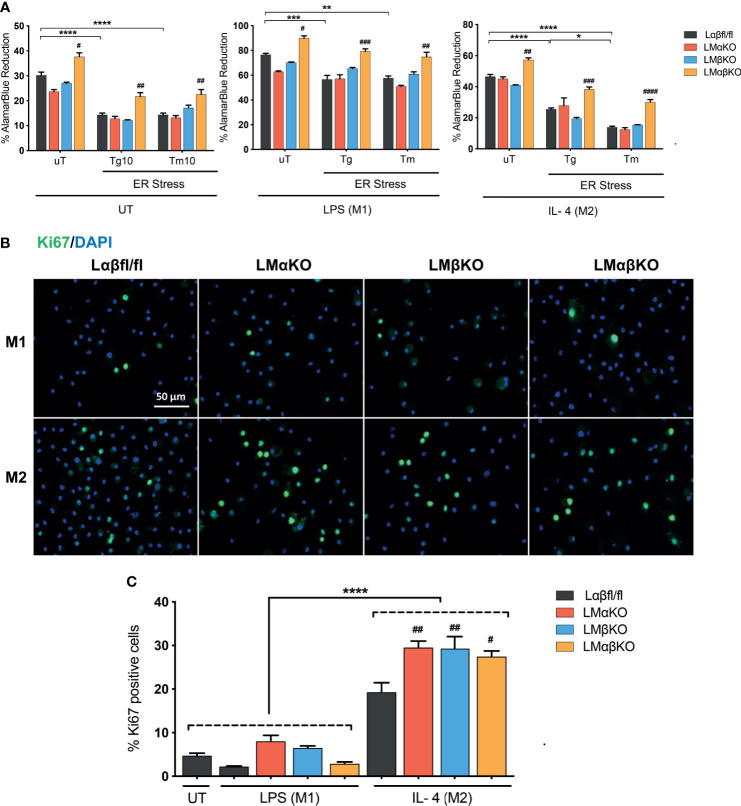
GSK3α and GSK3β have complementary roles in the regulation of macrophage viability. Cytotoxic effects of Tg or Tm was assessed by determining the cell viability of the macrophages. **(A)** Cell viability was quantified by normalizing the reduced alamarBlue values to untreated control. n=4-8. Proliferation of these macrophages were determined by **(B)** Ki67 staining of proliferated macrophages and proliferated cells were quantified by counting **(C)** the no. of Ki67 positive cells. n=4; mean ± SEM; * is the comparison between UT, LPS(M1), and IL-4(M2) treatments; # is the comparison between control and KOs (within same treatment); ^#^p < 0.05, ^##^p < 0.01, ^###^p < 0.001, ^####^p < 0.0001, *p < 0.05, **p < 0.01, *** p< 0.001, ****p < 0.0001. GSK3 indicates glycogen synthase kinase 3; UT, untreated; LPS, lipopolysaccharide; IL-4, interleukin-4; Tg, thapsigargin; Tm, tunicamycin; ER, endoplasmic reticulum.

We next determined the effect of GSK3α and/or GSK3β deficiency on macrophage viability and function. BMDM were challenged with ER stress inducing agents, tunicamycin (Tm) or thapsigargin (Tg). Cell viability (% alamarBlue reduction) was measured using an alamarBlue cell viability assay (**Figure 5A**). As expected, Lαβfl/fl (control) M1 and M2 macrophages show a decrease in alamarBlue reduction when treated with Tm or Tg. The alamarBlue reduction in LMαKO and LMβKO macrophages was not significantly different than the Lαβfl/fl (control). The alamarBlue reduction was significantly increased in both M1 and M2 macrophages that were deficient in both GSK3α and GSK3β. These results suggest that GSK3α and GSK3β play redundant roles in regulating the metabolic activity of macrophages.

Immunofluorescence staining was used to analyze the effects of GSK3α and/or GSK3β deficiency on macrophage proliferation markers. BMDM from Lαβfl/fl (control), LMαKO, LMβKO, and LMαβKO mice were fixed and immunostained with an antibody against the proliferation marker Ki67 and DAPI (**Figure 5B**) and quantified (**Figure 5C** and **Supplementary Figure VIIIA**). The percentage of Ki67 positive cells was significantly increased in M2 macrophages in comparison to M1 macrophages (**Figure 5C**). LMαKO, LMβKO, and LMαβKO M2 macrophages had significantly increased expression of Ki67, compared to Lαβfl/fl controls. There was no significant difference found in the gene expression of proliferation marker cMyc (**Supplementary Figure VIIIB**). These results suggest that GSK3α and GSK3β suppress M2 macrophage proliferation.

### Migration of M1 Macrophages Increase With GSK3α Deficiency and Decreases With GSK3β Deficiency

**Figure 6:**
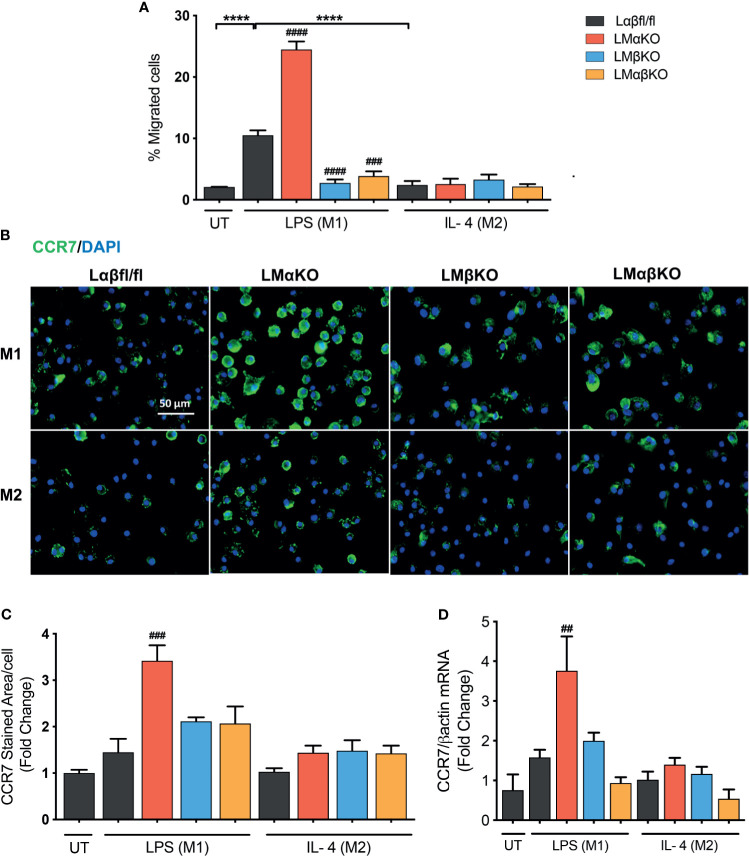
Migration of M1 macrophages enhanced with a GSK3α deficiency and impaired with a GSK3β deficiency. Migration of these macrophages towards CCL19 were determine by using transwell plates. **(A)** migrated cells were quantified by counting the no. of cells migrated. **(B)** CCR7 staining of GSK3α- and/or GSK3β-deficient macrophages and CCR7 expression was quantified by measuring **(C)** CCR7 stained area/celI. **(D)** Gene expression of CCR7 was measured in GSK3α-and/or GSK3β-deficient macrophages. Results are reported as the fold change relative to control UT. n=4; mean ± SEM; * is the comparison between M0, M1, and M2; # is the comparison between control and KOs (within same treatment); ^##^p < 0.01, ^###^p < 0.001, ^####^p < 0.0001, ****p < 0.0001. GSK3 indicates glycogen synthase kinase 3; UT, untreated; LPS, lipopolysaccharide; IL-4, interleukin-4; CCR7, C-C chemokine receptor type 7.

Macrophage migration was determined using a transwell plate assay. BMDM isolated from Lαβfl/fl (control), LMαKO, LMβKO, and LMαβKO mice were induced to migrate toward the chemokine, CCL19. As expected, M1 macrophages showed increased migratory activity towards CCL19, compared to M2 macrophages (**Figure 6A**). The results indicate that LMαKO macrophages had a greater tendency to migrate, whereas LMβKO and LMαβKO macrophages showed decreased migration.

To investigate the underlying mechanism, the effect of GSK3α and/or GSK3β deficiency on C-C chemokine receptor type 7 (CCR7) expression was examined (**Figures 6B–D**). M1 and M2 macrophages have similar levels of CCR7 protein (**Figures 6B, C**) and gene expression (**Figure 6D**). GSK3α-deficient macrophages displayed increased expression of CCR7 in M1 macrophages (**Figures 6C, D**). LMβKO and LMαβKO macrophages displayed no difference in expression of CCR7 in M1 or M2 macrophages (**Figures 6C, D**). These results suggest that GSK3α actively suppresses the expression of CCR7 expression. Analysis of other factors involved in macrophage migration, including sphingosine-1-phosphate receptor (S1PR) 1, S1PR3, and macrophage migration inhibitory factor (MIF), revealed no significant differences in gene expression (**Supplementary Figure IX**). Together these results suggest that GSK3α inhibits migration and GSK3β induces migration of M1 macrophages towards CCL19.

### Combined GSK3α and GSK3β Deficiency Increases Metabolic Activity

**Figure 7:**
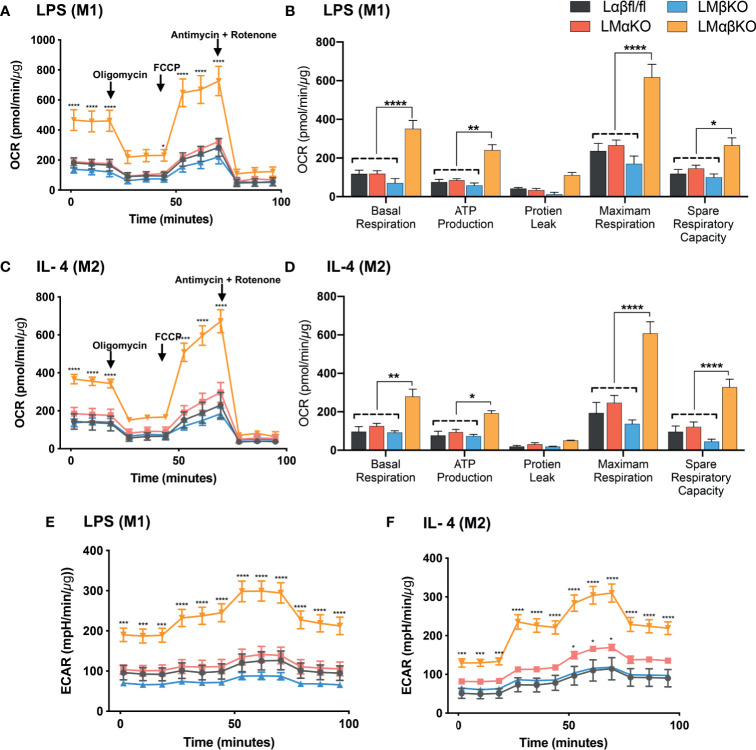
GSK3α and GSK3β both together play a role metabolic activity of macrophages Metabolic activity of macrophages were determine by using seahorse extracellular flux analysis. OXPHOS was measure by analyzing OCR (normalized to protein content) in GSK3α- and/or GSK3β-deficient **(A, B)** M1 macrophages and **(C, D)** M2 macrophages. Glycolysis was measure by analyzing ECAR (normalized to protein content) in GSK3α-and/or GSK3β-deficient **(E)** M1 macrophages and **(F)** M2 macrophages. n = 4; mean ± SEM; * is the comparison between control and KOs; *p < 0.05, **p < 0.01, ***p < 0.001, ****p < 0 .0001. GSK3 indicates glycogen synthase kinase 3; OXPHOS, oxidative phosphorylation; OCR, oxygen consumption rate; ECAR, extracellular acidification rate.

Metabolic activity was determined by measuring the change in oxidative phosphorylation in BMDM using the Seahorse Analyzer XF24. The XF Cell Mito Stress analysis was used to measure mitochondrial activity. BMDM from Lαβfl/fl (control), LMαKO, LMβKO, and LMαβKO mice were cultured for 24 hrs after which mitochondrial activity was measured (**Figure 7**). As expected, M2 macrophages showed increased oxygen consumption rate (OCR) in comparison to M1 macrophages (**Supplementary Figure XA**) and M1 macrophages showed increased extracellular acidification rate (ECAR) in comparison to M2 macrophages (**Supplementary Figure XB**). Results suggest that LMαβKO macrophages have an increased OCR (**Figures 7A, C**) and ECAR (**Figures 7E, F**) compared to Lαβfl/fl (control) in both M1 and M2 macrophages. LMαβKO macrophages show a significant increase in basal, ATP-linked, spare respiratory capacity, and maximal OCR (**Figures 7B, D**). LMαKO and LMβKO macrophages showed no change in OCR in both M1 and M2 macrophages (**Figures 7A, C**). Together these results suggest that GSK3α and GSK3β play redundant roles in regulating metabolic activity.

## Discussion

**Figure 8:**
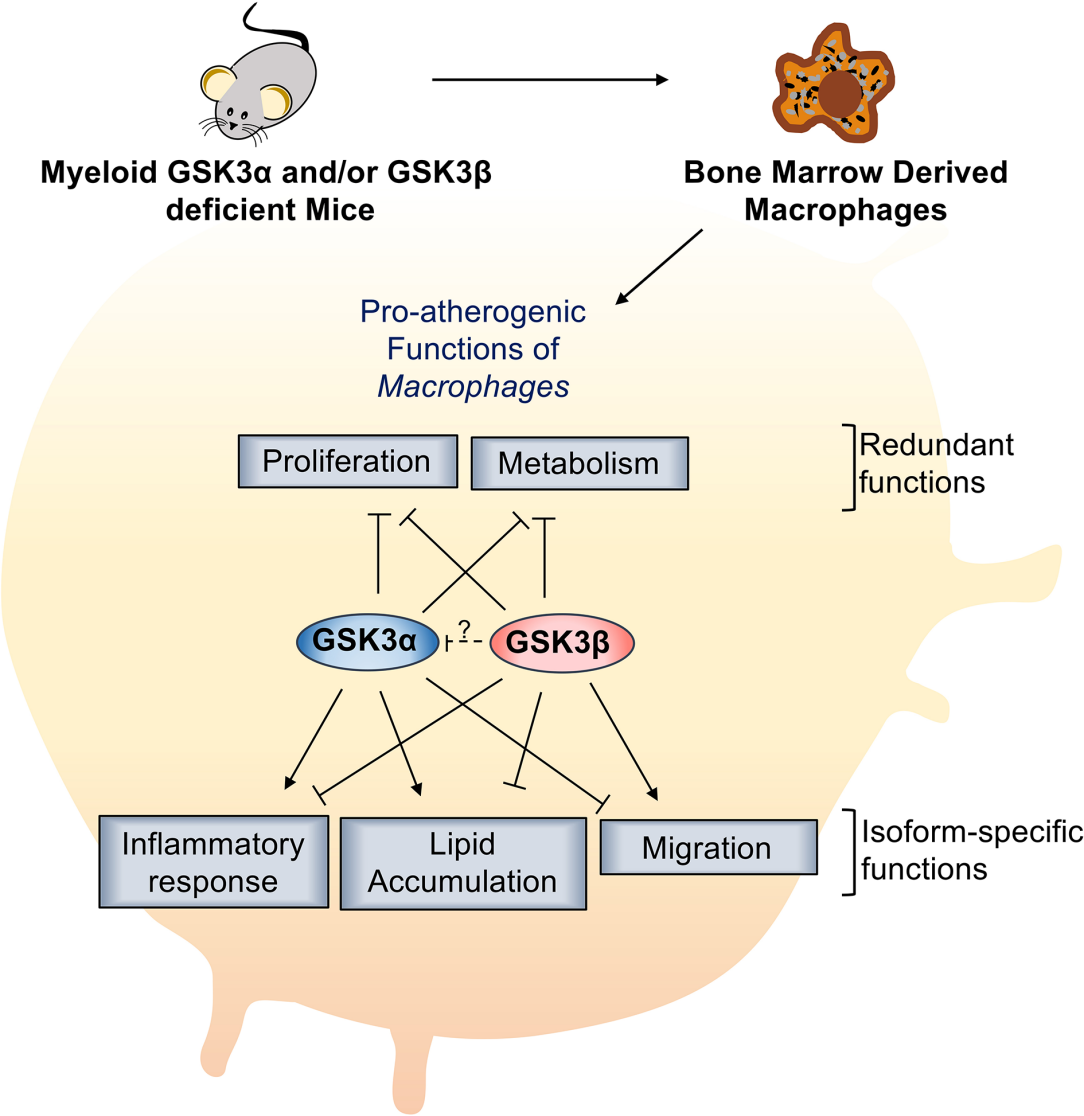
Specific role of myeloid GSK3α/β in proatherogenic functions. Myeloid GSK3α and GSK3β have opposing roles in macrophage polarization, inflammatory response, lipid accumulation, and migration of macrophages. Myeloid GSK3α and GSK3β play redundant roles to increase macrophage(M2) proliferation and metabolism of macrophages.

GSK3α and GSK3β are highly homologous, constitutively active kinases that are expressed in most cells including macrophages. GSK3α and GSK3β function within central signal transduction pathways that regulate cell viability and metabolism and over 100 putative substrates have been identified (17). Dysregulation of GSK3α/β has been implicated in several metabolic disorders. Recent evidence suggests that GSK3α and GSK3β play distinct roles in sperm motility and fertility (35), amyloid production in the brain (13), cortical development (36), atherosclerosis development (37), and acute myeloid leukemia development (38). However, therapeutic targeting has been limited by our lack of understanding of homolog-specific functions as well as the lack of isoform-specific inhibitors. Here, we delineate the specific roles of GSK3α and GSK3β in macrophage polarization and pro-atherogenic functions. We show that myeloid GSK3α and GSK3β have distinct effects in the regulation of macrophage phenotype, inflammatory response, lipid accumulation, and migration (**Figure 8**). Conversely, GSK3α and GSK3β appear to play complementary, redundant roles in macrophage viability, proliferation, and metabolism (**Figure 8**).

The mechanisms regulating macrophage polarization involve specific signaling pathways. In M1 polarization, LPS and IFN-γ interact with cell surface receptors to promote the activation of NF-κB and hypoxia-inducible factor 1-alpha (HIF1α), which induce expression of pro-inflammatory cytokines (TNFα, IL-1β) and iNOS (38). Our results suggest that GSK3α-deficient macrophages exhibit impaired LPS-induced gene expression of iNOS, STAT1 and CD38, resulting in impaired M1 polarization. This is consistent with our previous findings showing that GSK3α deletion suppresses M1 polarization in macrophages within the atherosclerotic lesion (29, 30). GSK3β-deficient macrophages have increased LPS-induced gene expression of iNOS and STAT1. GSK3β-deficient macrophages also have decreased IL-4-induced Arg1, Fizz1 and Ym1 expression, resulting in impaired M2 polarization. Together, these data are consistent with a pro-inflammatory role for GSK3α, and an anti-inflammatory role for GSK3β during macrophage polarization.

Previous reports have suggested both pro- and anti-inflammatory roles for GSK3β. Specifically, GSK3β has been shown to negatively regulate TLR4-mediated pro-inflammatory cytokine (IFN-β) production (39). In neonatal mouse cardiomyocytes and heart tissue culture, inhibition of GSK3β with chemical and genetic inhibitors enhanced LPS-induced pro-inflammatory cytokine expression (40). GSK3β has been shown to be capable of both activating and inhibiting NF-κB (41). GSK3β-deficient embryos have reduced NF-κB activity (24). Signaling through the ANO-PI3K–Akt pathway, which inhibits GSK3β, leads to increased binding of the cAMP response element-binding protein (CREB) to nuclear coactivator CREB-binding protein (CBP), resulting in suppression of the binding of NF-κB p65 to CBP (42). Another study suggests that inactivation of GSK3β *via* PI3K-Akt signaling pathway enhanced NF-κB activity, which leads to subsequent production of pro-inflammatory cytokines (43). Much less is known about the ability of GSK3α to modulate inflammation.

Our result suggests that GSK3α-deficient macrophages exhibit impaired LPS-induced expression of NF-κB. GSK3β-deficient macrophages have increased LPS-induced expression of NF-κB and NLRP3. This result is consistent with previous reports showing the inactivation of GSK3β enhanced NF-kB activity and increases the inflammatory response (44). Furthermore, our results show that GSK3α deficiency decrease the production of pro-inflammatory cytokines/chemokines (IL-1α, IL-1β, TNFα, IL-6, IL-12p70, MCP-1, MIP-2, KC) and GSK3β deficiency increase the production of pro-inflammatory cytokines/chemokines (IL-1α, IL-1β, MCP-1, MIG). This suggest that GSK3α positively- and GSK3β negatively- regulate NF-kB to induce pro-inflammatory response in LPS treated (M1) macrophages. Together, these results suggest that GSK3α and GSK3β function to regulate M1 and M2 polarization, with GSK3α promoting the pro-inflammatory response and GSK3β supporting the anti-inflammatory response in macrophages.

Macrophages endocytose modified-LDL particles through the action of scavenger receptors (SR-A and CD36) and become foam cells. Consistent with previous reports (45), we found that M1 macrophages accumulate significantly more lipid compared to M2 macrophages. Furthermore, we show that lipid accumulation in M1 macrophages is reduced with GSK3α deficiency and increased with GSK3β deficiency. To determine the mechanism(s) underlying these observations we assessed the expression levels of genes involved in lipid uptake, export, and biosynthesis (46–49). We found increased expression of genes encoding SR A and CD36 in GSK3β- and GSK3αβ-deficient macrophages. This suggests that there is an overall increase in lipid transport in GSK3β-deficient macrophages that results in increased lipid accumulation. Furthermore, we found increased gene expression of ABCA1 and ABCG1 in GSK3β- and GSK3αβ double-deficient macrophages. This suggests that the lipid accumulation leads to increased expression of genes involved in lipid efflux. Together, these findings suggest that GSK3α promotes lipid accumulation and GSK3β impedes lipid accumulation in M1 macrophages. The observation that GSK3β-deficient and GSK3αβ double-deficient macrophages have similar phenotypes may indicate that GSK3α is regulated by GSK3β.

Our previously published findings have suggested that cardiovascular risk factors promote atherogenesis by a mechanism involving ER stress-induced activation of GSK3α/β (50, 51). This pathway promotes lipid accumulation and apoptosis of macrophage/foam cells *in vitro (* 19). We investigated the effect of ER stress on macrophage viability in GSK3α- or GSK3β-deficient cells. We found that myeloid deficiency of either the GSK3α or GSK3β isoform did not have any effect on cell viability. Deficiency of both isoforms significantly increased cell viability in both M1 and M2 macrophages. These results suggest that GSK3α and GSK3β are both required to facilitate ER stress-induced cell death pathways.

It has recently become evident that lesional macrophage proliferation may play an important role in atherosclerotic plaque development (52, 53). The complete understanding of the molecular and cellular mechanisms that regulate macrophage proliferation in atherosclerotic lesions is still unknown. Our results show that there were significantly more Ki67 positive cells when macrophages were polarized to an M2 phenotype relative to an M1 phenotype. A previous study has shown that exposure to Th2 cytokines stimulates adipose tissue macrophage (M2) proliferation and inhibits M1 proliferation (54). Deficiency of GSK3α and/or GSK3β significantly increases the % of Ki67 positive cells. These results suggest that GSK3α and/or GSK3β suppress M2 macrophage proliferation.

To effectively carry out their phagocytic role within the artery wall, macrophages are required to migrate toward chemotactic signals within the lesional environment. Macrophage migration is known to be regulated by several factors including intracellular cholesterol content (55–57). We investigated the effect of GSK3α and/or β deficiency on CCL19-CCR7 stimulated migration using a transwell assay (58). Our results show that GSK3α-deficient macrophages are more proficient at migrating, whereas GSK3β- and GSK3αβ double-deficient macrophages are less proficient at migrating, towards a CCL19 chemokine signal. To determine if migration ability was regulated at the level of chemokine receptor expression, we determined the expression of CCR7 in these GSK3α/β-deficient macrophages (58). As expected, we found that both M1 and M2 macrophages have similar CCR7 expression levels. GSK3α-deficient macrophages, but not GSK3β-deficient macrophages, had increased expression of CCR7 in M1 macrophages. Together, these results suggest that GSK3α inhibits the migration of M1 macrophages towards CCL19 by regulating CCR7 expression.

M1 macrophages are known to have enhanced glycolytic metabolism and reduced mitochondrial activity, whereas M2 macrophages rely upon mitochondrial oxidative phosphorylation (OXPHOS). The effect of metabolic changes in macrophages on the development of atherosclerotic plaque is poorly defined. Recent evidence suggests that a high degree of anaerobic glycolysis (59) and mitochondrial oxidative stress (60) in macrophages play an important role in the development of advanced atherosclerosis lesions. Our results indicate that deficiency of both GSK3α and GSK3β increases the mitochondrial activity (OCR) in both M1 and M2 macrophages. These results suggest that GSK3α and GSK3β play a redundant role in the mitochondrial activity of macrophages.

It is important to note that both the control BMDM and the GSK3α and/or GSK3β-deficient BMDM utilized in these experiments come from Ldlr-deficient mouse strains. In addition to modulating circulating lipid levels, the Ldlr is known to indirectly effect inflammatory responses and cellular metabolism. At the present time, we do not know how the presence or absence of the Ldlr might affect these results.

In summary, GSK3α and GSK3β play distinct, and often opposing roles in the signaling pathway of atherogenic functions. Consistent with previous findings, myeloid GSK3α signaling appears to play an important pro-atherogenic role, inducing M1 macrophage polarization and activating pro-atherogenic pathways to accelerate plaque development. Further investigations are needed to delineate the downstream substrates and pathways through which GSK3α acts and to explore the potential for targeting GSK3α with isoform specific small molecule inhibitors as an anti-atherogenic therapy.

## Data Availability Statement

The original contributions presented in the study are included in the article/**Supplementary Material**. Further inquiries can be directed to the corresponding author.

## Author Contributions

GW and SP conceived and designed the study. SP conducted and analyzed all experiments except Extracellular Flux assay. All authors contributed to the article and approved the submitted version.

## Funding

This research was supported by operational grants from the Canadian Institutes for Health Research (CIHR, PJT-166092) and Heart and Stroke Foundation of Canada (HSFC, G20-0029355). GW holds the ISTH-McMaster Chair in Thrombosis and Haemostasis Research and is supported by a HSFC Ontario Mid-Career Investigator Award.

## Conflict of Interest

The authors declare that the research was conducted in the absence of any commercial or financial relationships that could be construed as a potential conflict of interest.

## Publisher’s Note

All claims expressed in this article are solely those of the authors and do not necessarily represent those of their affiliated organizations, or those of the publisher, the editors and the reviewers. Any product that may be evaluated in this article, or claim that may be made by its manufacturer, is not guaranteed or endorsed by the publisher.
