# Supplementary material for: Characterizing the Role of Glycogen Synthase Kinase-3α/β in Macrophage Polarization and the Regulation of Pro-Atherogenic Pathways in Cultured Ldlr-/- Macrophages
Source: Front Immunol. 2021 Jul 30;12:676752. doi: 10.3389/fimmu.2021.676752 (PMC8361494; doi:10.3389/fimmu.2021.676752)
Supplement: Supplementary file 1 [file DataSheet_1.docx]

Supplementary Material

# Supplementary Data

**Genetically Modified Animals**

| **Background Strain** | **Species** | **Genotype** | **Deficiency (Ldlr^-/-^)** |
| --- | --- | --- | --- |
| **C57BL/6J** | **LMαβfl/fl** | *Ldlr^-/-^GSK3α^fl/fl^GSK3β^fl/fl^* | Control |
|  | **LMαKO** | *Ldlr^-/-^GSK3α^fl/fl^LysMCre^+/-^* | myeloid GSK3α^-/-^ |
|  | **LMβKO** | *Ldlr^-/-^GSK3β^fl/fl^LysMCre^+/-^* | myeloid GSK3β^-/-^ |
|  | **LMαβKO** | *Ldlr^-/-^GSK3α^fl/fl^GSK3β^fl/fl^LysMCre^+/-^* | myeloid GSK3α^-/-^β^-/-^ |

**Antibodies used**

| **Target antigen** | **Vendor or Source** | **Working concentration** | **Experiment** |
| --- | --- | --- | --- |
| CD16/32 | eBioscience | 1:100 | Flow Cytometry |
| CD11b-APC | Life Technologies | 1:50 | Flow Cytometry |
| F4/80-PE | BD Pharmagen | 1:50 | Flow Cytometry |
| GSK-3α/β | Cell signaling | 1:1000 | WB |
| GSK-3β | Cell signaling | 1:1000 | WB |
| β-Actin | SIGMA | 1:3000 | WB |
| Ki67 | Abcam | 1:200 | IF |
| NFKβ | Santa Cruz | 1:50 | IF |
| NLRP3 | Abcam | 1:50 | IF |
| CCR7 | Abcam | 1:100 | IF |

# Supplementary Figures and Tables

**Supplementary Table I.** RT-PCR primer sequences


**Supplementary Table II.** Complete blood cell count

**Supplementary Figure I. Negative Controls for immunofluorescent staining***.* BMDMs were stained with pre-immune IgG antibodies to control for non-specific binding. Representative images of control IgG immunofluorescent staining and specific secondary antibody staining for **A.** anti-mouse, **B.** anti-rabbit. DAPI nuclear counterstaining is shown in blue. NC, negative control.

**Supplementary Figure II.** Uncropped Images of western blot analysis of BMDM.

**Supplementary Figure III.** **Effect of GSK3α and/or GSK3β deficiency on macrophage differentiation.** To determine the bone marrow progenitor cells differentiation into macrophages, cells were labelled with antibodies against the macrophage-specific surface markers, CD11b and F4/80. Cells from each experimental group were examined on a BD FACS calibur flow cytometer. Representative contour diagram of CD11b/F4/8 of BMDM from **A.** GSK3α deficient mice**, B.** GSK3β deficient mice and **C.** GSK3α and GSK3β deficient mice in compare to their respective control mice.

**Supplementary Figure IV. Macrophage polarization.** Polarization efficiency were examined by quantify the transcription expression of the gene associated with M1, M2 and Mox macrophage polarization by using RT-PCR. **A.** M1 polarization (iNOS, STAT1, CD38) **B.** M2 polarization (Arg1, Fizz1, Ym1) **C.** Mox polarization (HO1, Txnd1). Data presented in Figure 2 with the addition of effects on M0 macrophages. Data are normalized to the βactin reference gene. Results are reported as the fold change relative to control M0. n=3-4; mean ± SEM; * is the comparison between UT, LPS(M1), and IL-4(M2) treatments; # is the comparison between control and KOs (within same treatment); *p<0.05, **p<0.01, ****p*<0.001, *****p*<0.0001.

**Supplementary Figure V. Effect of GSK3α and/or GSK3β deficiency on transcription factors expression involved in inflammation.** Expression of transcription factors were examined by quantify gene expression using RT-PCR. **A.** C/EBPβ **B.** CREB. Data are normalized to the βactin reference gene. Results are reported as the fold change relative to control M0. n=3-4; mean ± SEM; * is the comparison between UT, LPS(M1), and IL-4(M2) treatments; # is the comparison between control and KOs (within same treatment); *p<0.05, *****p*<0.0001.

**Supplementary Figure VI. Effect of GSK3α and/or GSK3β deficiency on anti-inflammatory cytokine production.** Concentration of anti-inflammatory cytokines (IL-10 and IL-4) in culture media were determined by mouse cytokine and chemokine array after 24 hours of UT or IL-4(M2) treatment. n=3-4; mean ± SEM; * is the comparison between UT and IL-4(M2) treatment; # is the comparison between control and KOs (within same treatment); *p<0.05, **p<0.01, ****p*<0.001, *****p*<0.0001.

**Supplementary Figure VII. Effect of GSK3α and/or GSK3β deficiency on lipid accumulation and metabolism.** Lipid accumulation of these macrophages were determined by Oil Red O staining. **A.** Oil red O staining of GSK3α and/or GSK3β deficient macrophages. Transcription expression of the gene associated with lipid accumulation and metabolism were quantified **B.** SR B1 **C.** LXRα **D.** LCAT **E.** HMG CoA **F.** FAS. Results are reported as the fold change relative to control UT. n=3-4; mean ± SEM; * is the comparison between UT, LPS(M1), and IL-4(M2) treatments; # is the comparison between control and KOs (within same treatment); *p<0.05, **p<0.01, ****p*<0.001, *****p*<0.0001.


**Supplementary Figure VIII. Effect of GSK3α and/or GSK3β deficiency on proliferation**. **A.** Proliferation of macrophages were determined by Ki67 staining and proliferated cells were quantified by counting the no. of Ki67 positive cells. Transcription expression of the gene associated with proliferation were quantified **B.** cMyc. Results are reported as the fold change relative to control UT. n=3-4; mean ± SEM; * is the comparison between UT, LPS(M1), and IL-4(M2) treatments; # is the comparison between control and KOs (within same treatment); *p<0.05, **p<0.01, ****p*<0.001, *****p*<0.0001.

**Supplementary Figure IX. Effect of GSK3α and/or GSK3β deficiency on migration.** Transcription expression of the gene associated with migration were quantified **A.** S1PR1 **B.** S1PR3 **C.** MIF. Results are reported as the fold change relative to control UT. n=3-4; mean ± SEM; * is the comparison between UT, LPS(M1), and IL-4(M2) treatments; # is the comparison between control and KOs (within same treatment); *p<0.05, **p<0.01, ****p*<0.001, *****p*<0.0001.

**Supplementary Figure X. Metabolism in M1 and M2 polarized BMDM.** Metabolic activity of macrophages were determined by using seahorse extracellular flux analysis. **A.** OXPHOS was measure by analysing OCR (normalized to protein content) and **B**. Glycolysis was measure by analysing ECAR (normalized to protein content) in control BMDM. n=4; mean ± SEM.
